# Modeling Optimal Laboratory Testing Strategies for Bacterial Meningitis Surveillance in Africa

**DOI:** 10.1093/infdis/jiab154

**Published:** 2021-09-01

**Authors:** Joseph Walker, Heidi M Soeters, Ryan Novak, Alpha Oumar Diallo, Jeni Vuong, Brice Wilfried Bicaba, Isaie Medah, Issaka Yaméogo, Rasmata Ouédraogo-Traoré, Kadidja Gamougame, Daugla Doumagoum Moto, Assétou Y Dembélé, Ibrehima Guindo, Souleymane Coulibaly, Djibo Issifou, Maman Zaneidou, Hamadi Assane, Christelle Nikiema, Adodo Sadji, Katya Fernandez, Jason M Mwenda, Andre Bita, Clément Lingani, Haoua Tall, Félix Tarbangdo, Guetwende Sawadogo, Marietou F Paye, Xin Wang, Lucy A McNamara

**Affiliations:** 1 Department of Epidemiology, College of Public Health, University of Georgia, Athens, Georgia, USA; 2 Division of Bacterial Diseases, National Center for Immunization and Respiratory Diseases, Centers for Disease Control and Prevention, Atlanta, Georgia, USA; 3 Direction de la Protection de la Santé de la Population, Ouagadougou, Burkina Faso; 4 Centre Hospitalier Universitaire Pédiatrique Charles De Gaulle, Ouagadougou, Burkina Faso; 5 Ministère de la Santé Publique du Tchad, N’Djamena, Tchad; 6 Swiss Tropical and Public Health Institute, N’Djamena, Tchad; 7 Direction Nationale de la Santé, Bamako, Mali; 8 Institut National de Recherche en Santé Publique, Bamako, Mali; 9 Direction de la Surveillance et Riposte aux Epidémies, Ministère de la Santé Publique, Niamey, Niger; 10 Ministère de la Santé et de l’Hygiène Publique, Lomé, Togo; 11 Institut National D’Hygiène, Lomé, Togo; 12 World Health Organization Infectious Hazard Management, Geneva, Switzerland; 13 World Health Organization Regional Office for Africa, Brazzaville, Congo; 14 World Health Organization Inter-Country Support Team West Africa, Ouagadougou, Burkina Faso; 15 Agence de Médecine Préventive, Ouagadougou, Burkina Faso; 16 Davycas International, Ouagadougou, Burkina Faso; 17 Centers for Disease Control and Prevention Foundation, Contracted to Division of Bacterial Diseases, National Center for Immunization and Respiratory Diseases, Centers for Disease Control and Prevention, Atlanta, Georgia, USA; 18 Influenza Division, National Center for Immunization and Respiratory Diseases, Centers for Disease Control and Prevention, Atlanta, Georgia; 19 Global Immunization Division, Center for Global Health, Centers for Disease Control and Prevention, Atlanta, Georgia; 20 Department of Epidemiology, University of North Carolina at Chapel Hill, Chapel Hill, North Carolina; 21 Division of Global HIV & TB, Center for Global Health, Centers for Disease Control and Prevention, Atlanta, Georgia

**Keywords:** Bacterial Meningitis, Laboratory Surveillance, Modeling, Burkina Faso, Chad, Mali, Niger, Togo

## Abstract

Since 2010, the introduction of an effective serogroup A meningococcal conjugate vaccine has led to the near-elimination of invasive *Neisseria meningitidis* serogroup A disease in Africa’s meningitis belt. However, a significant burden of disease and epidemics due to other bacterial meningitis pathogens remain in the region. High-quality surveillance data with laboratory confirmation is important to monitor circulating bacterial meningitis pathogens and design appropriate interventions, but complete testing of all reported cases is often infeasible. Here, we use case-based surveillance data from 5 countries in the meningitis belt to determine how accurately estimates of the distribution of causative pathogens would represent the true distribution under different laboratory testing strategies. Detailed case-based surveillance data was collected by the MenAfriNet surveillance consortium in up to 3 seasons from participating districts in 5 countries. For each unique country-season pair, we simulated the accuracy of laboratory surveillance by repeatedly drawing subsets of tested cases and calculating the margin of error of the estimated proportion of cases caused by each pathogen (the greatest pathogen-specific absolute error in proportions between the subset and the full set of cases). Across the 12 country-season pairs analyzed, the 95% credible intervals around estimates of the proportion of cases caused by each pathogen had median widths of ±0.13, ±0.07, and ±0.05, respectively, when random samples of 25%, 50%, and 75% of cases were selected for testing. The level of geographic stratification in the sampling process did not meaningfully affect accuracy estimates. These findings can inform testing thresholds for laboratory surveillance programs in the meningitis belt.

Bacterial meningitis is a deadly disease with case fatality ratios that can reach 70% without rapid treatment; up to 20% of survivors experience persistent physical or cognitive disability [1]. Globally, the most common causes of bacterial meningitis are *Neisseria meningitidis*, *Streptococcus pneumoniae*, and *Haemophilus influenzae* [2, 3], with the highest incidence of disease observed in Africa’s “meningitis belt,” the semi-arid region south of the Saharan Desert [4]. Meningitis dynamics are highly seasonal within the belt with the period of highest risk coinciding with the dry season, generally extending from December to June. In addition to seasonal outbreaks of disease, the belt also intermittently experiences larger epidemics driven by the circulation of highly invasive pathogen strains [5].

Historically, most epidemics of meningitis in the belt were caused by *N. meningitidis* serogroup A (NmA) [6]. Between 2010 and 2018, meningococcal A conjugate vaccine (MACV) was introduced in 22 of the 26 countries in the meningitis belt through mass vaccination campaigns immunizing close to 300 million people aged 1–29 years of age. As of the end of 2018, 8 of the countries that conducted a mass campaign have also introduced MACV as a routine childhood vaccination through the Expanded Program on Immunization [7]. As a result of the widespread rollout of MACV, serogroup A meningococcal disease has been nearly eliminated from the meningitis belt [8–10]. However, other pathogens have emerged as the primary causes of bacterial meningitis outbreaks and disease in the belt, including *N. meningitidis* serogroups C, W, and X and *S. pneumoniae*.

The MenAfriNet Consortium has partnered with the Ministries of Health of Burkina Faso, Chad, Mali, Niger, and Togo since 2014 to implement meningitis case-based surveillance [11]. Efficient laboratory confirmation is a critical component of meningitis case-based surveillance to monitor epidemiologic trends of circulating bacterial meningitis pathogens, evaluate existing vaccines and other public health interventions, and inform policy decisions and development of new vaccines. However, specimen collection, transport, and testing all require public health and laboratory resources that are in limited supply in many parts of the meningitis belt. Thus determining the minimum level of specimen collection and laboratory testing necessary for public health action could help to conserve and allocate limited resources. In this analysis, we use case-based surveillance data from the 5 MenAfriNet countries to model the expected accuracy of pathogen-specific meningitis burden estimates at different levels of laboratory testing.

## METHODS

### Surveillance System

This analysis used 2014–2017 MenAfriNet meningitis case-based surveillance data [11]. Participating ministries of health collect case-level demographic, clinical, and laboratory data from cerebrospinal fluid (CSF) specimens for meningitis cases in selected districts. By 2017, case-based meningitis surveillance through MenAfriNet covered approximately 32.7 million people living in all districts in Burkina Faso and 115 (33%) of 347 districts in Chad, Mali, Niger, and Togo [12]. Bacterial meningitis cases are classified using case definitions established by the World Health Organization [13]. Under these criteria, suspected cases are distinguished by a sudden onset of fever above 38.5°C with ≥1 meningeal sign, such as neck stiffness, convulsions, or bulging fontanelle. For a case to be classified as confirmed, the criteria for a suspected case must be met and *N. meningitidis*, *S. pneumoniae, H. influenzae*, or another bacterial meningitis pathogen must be identified in CSF by culture or real-time polymerase chain reaction (PCR).

CSF specimens were collected from each case and transported to a national reference laboratory (NRL), either directly or first through the district and/or regional public health laboratory, either in transisolate medium at room temperature for culture and/or in a cryotube via cold chain for real-time PCR. At the NRL, culture, latex agglutination, and/or PCR testing were used to attempt to identify one of the following meningitis-associated bacterial pathogens: (1) *N. meningitidis,* serogroups A, B, C, W, X, or Y; (2) *S. pneumoniae*; (3) *H. influenzae,* serotype b and non–serotype b; and (4) group B *Streptococcus.* PCR targeted the *sodC* gene for *N. meningitidis, lytA* for *S. pneumoniae*, and *hpd* for *H. influenzae*. Confirmed cases of group B *Streptococcus* were identified via culture.

A total of 2673 cases were tested with both culture and PCR. Of these, 957 (35.8%) were positive by one or both methods: 936 (35.0%) by PCR, 306 (11.4%) by culture, and 285 (10.7%) by both. In the 60 cases (2.2%) in which PCR and culture testing identified different pathogens, the PCR result was used because of its higher sensitivity, low contamination rate among PCR specimens in the MenAfriNet network [12], and implementation of external quality control to ensure reliability of PCR results. If culture was not performed at the NRL, the culture result reported from the district or regional laboratory was used, if available (167 of 3077 cases [5.4%] with a culture result). *N. meningitidis*–positive specimens for which serogroup could not be determined were classified as serogroup-indeterminate, a category which includes both non-ABCWXY serogroups and nongroupable (unencapsulated) strains of *N. meningitidis*.

### Statistical Analysis

Each case was assigned a date based on the day of consultation at the health center. If this field was missing, the date of CSF collection was used in its place. For cases missing both of these fields (n = 6), the date the specimen arrived at the NRL was used. We then grouped cases into distinct country-seasons, which we defined as the period from 1 November of one year to 31 August of the subsequent year in a specific country. We selected this period, which includes 97% and 99% of suspected and confirmed cases, respectively, on the basis that it would capture the typical meningitis season (approximately December–June) and seasons with unusually early or late timing [4, 14, 15]. 

**Figure 1.**
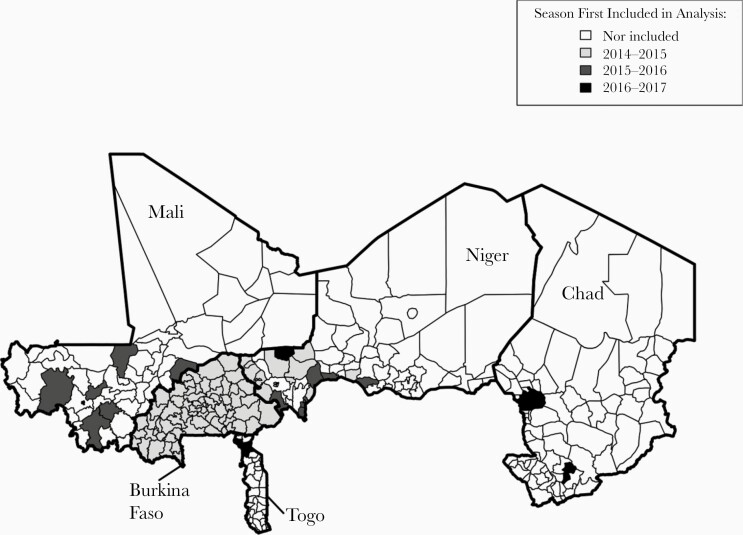
Map of health districts by first season of inclusion in analysis.

Cases were included only if they were reported from a district participating in MenAfriNet, and country-seasons containing <30 confirmed cases were excluded from the analysis (Figure 1). Suspected meningitis cases with specimens that underwent culture and/or PCR testing at a NRL were classified as either confirmed or unconfirmed based on the above case definition. For each confirmed case, we defined the causative pathogen as the pathogen species and serogroup/serotype (where applicable) identified by PCR (n = 3959), or by culture if PCR results were unavailable or negative (n = 59). For this analysis, *H. influenzae* serotype b and non–serotype b were treated as distinct pathogens, as were individual *N. meningitidis* serogroups (A, B, C, W, X, Y, and indeterminate)*. S. pneumoniae* and group B *Streptococcus* were each treated as a single pathogen in this analysis, as the serotypes of these pathogens were not routinely reported.

Using data on laboratory-tested cases, we sought to determine how accurately estimates of the distribution of causative pathogens would represent the true distribution under different laboratory testing strategies. To simulate the distributions of causative pathogens that may be observed with less-complete testing, we selected subsets of cases with CSF specimens tested via PCR or culture (tested cases) from each country-season using both a random- and a sequential-sampling testing strategy. Under the random-sampling testing strategy, we constructed 3 geographic sampling levels or strata by generating subsets of tested cases at the country, region, and district level. We formed the country sampling level by randomly selecting *p%* of tested cases (testing coverage level) from a country-season. Similarly, we generated the region-stratified and district-stratified sampling levels by randomly selecting *p%* of tested cases from each region and each district of the country in a given season, respectively. If *p%* of tested cases was not an integer value, we rounded down to the nearest whole case. For each country-season, geographic-sampling level (unstratified, region-stratified, and district-stratified), and testing coverage level, ranging from *p = 5%* to *p = 100%* (5% increments), we selected 2000 random subsets of cases to allow error estimation (see below).

For a given country-season, the random-sampling strategy would be expected to provide an unbiased estimate of the true pathogen distribution among confirmed cases. In practice, however, timely outbreak identification and response requires that laboratory results be obtained and reported regularly over the course of the season [13], precluding selection of a truly random sample of specimens for testing. To assess the potential impact of nonrandom selection of cases for testing, we implemented 3 forms of sequential sampling (unstratified, region stratified, and district stratified) as complements to the random sampling analysis. Under sequential sampling, we selected the first *p%* of tested cases observed within each geographic stratum (the entire country-season, each region, and each district), with values of *p* on the interval from *p = 5%* to *p = 100%* (2.5% increments). Because the sequential sampling method was nonrandom, only a single subset was generated for each combination of country-season, geographic stratum, and testing coverage level.

For each country-season and sampling strategy, we first calculated the proportion of confirmed cases associated with each pathogen for each subset of tested cases (“estimated pathogen proportions”) as well as in the full set of tested cases (“true pathogen proportions”). Then we calculated the margin of error of pathogen proportion estimates (“margin of error”) for each subset, which we define as the greatest absolute error of the pathogen proportion estimates (relative to the true pathogen proportion) from a given subset. Thus, all pathogen proportion estimates from a subset are within the subset’s margin of error of the true values.

For each unique random sampling strategy, defined by the proportion of cases selected for testing and the level of geographic stratification, we defined 2 distinct margin of error summary values: (1) the *average margin of error,* calculated by averaging the absolute value of the margin of error across each of the 2000 subsets; and (2) the *95% credible margin of error,* calculated by taking the 95th percentile margin of error value across all 2000 random subsets. The latter provides an estimate of the level of accuracy that one could expect to achieve with 95% confidence for the random-sampling strategy.

For each sequential subset, we derived a single margin of error value, the *sequential margin of error,* defined as the greatest absolute difference in pathogen proportion values between the subset and the full set of cases. For each summary value, the median across the 12 included country-seasons was presented.

Documenting the presence or absence of rare pathogen strains in a population requires the collection of a sufficient number of representative samples—more than are needed to document comparatively common strains—efficient specimen transport, and quality laboratory testing. Pathogens of interest may include emerging strains as well as those that have been locally controlled or eliminated through vaccination, such as NmA after the mass rollout of MACV in meningitis belt countries. Using NmA in the Burkina Faso 2014–2015 season as a case study [16], we estimated the probability of detecting ≥1 of the 4 NmA cases that occurred in that country-season (the proportion of random subsets containing ≥1 NmA cases) for different levels of testing coverage and geographic stratification.

In addition to pathogen-specific measures, the overall proportion of tested cases that are confirmed as bacterial meningitis (test-positive proportion) is an important metric of meningitis activity and surveillance system functioning. To assess the accuracy of the test-positive proportion under different sampling strategies, we also calculated the absolute margin of error of the test-positive proportion in each subset at different levels of testing coverage and geographic stratification for each country-season.

Data were analyzed using R version 3.5.1. Analysis of data collected through routine MenAfriNet surveillance was determined by the Human Research Protection Office of the Centers for Disease Control and Prevention to be public health nonresearch, and institutional review board review was not required by any participating institutions.

## RESULTS

### Descriptive Epidemiology

Case-based surveillance data were available for 12 MenAfriNet country-seasons: the 2016–2017 season in Chad, the 2014–2015, 2015–2016, and 2016–2017 seasons in Burkina Faso, Niger, and Togo, and the 2015–2016 and 2016–2017 seasons in Mali. In total, 17 237 suspected cases of meningitis were reported in MenAfriNet districts during these seasons [Table 1]. Data from the 2014–2015 and 2015–2016 seasons were not available for Chad, which joined the MenAfriNet consortium in 2016. We did not include the 2014–2015 Mali season in this analysis because only 2 confirmed cases were reported during this season in MenAfriNet districts.

**Table 1.** Bacterial Meningitis Cases by Season and Country in MenAfriNet Data Set

Countries by Season	Suspected Cases, No.	Cases, No. (% of Tested Cases)		Cases, No. (% of Confirmed Cases)		
		Tested Cases	Confirmed Cases	*Neisseria meningitidis*	*Streptococcus pneumoniae*	*H. influenzae*
Burkina Faso						
2014–2015	2454	1795 (73.1)	741 (41.3)	262 (35.4)	453 (61.1)	26 (3.5)
2015–2016	2427	1805 (74.4)	683 (37.8)	161 (23.6)	484 (70.9)	38 (5.6)
2016–2017	2512	1751 (69.7)	559 (31.9)	174 (31.1)	353 (63.1)	32 (5.7)
Chad						
2016–2017	120	116 (96.7)	54 (46.6)	22 (40.7)	26 (48.1)	6 (11.1)
Mali						
2015–2016	290	262 (90.3)	71 (27.1)	26 (37.1)	35 (50.0)	9 (12.9)
2016–2017	240	214 (73.8)	47 (22.0)	6 (12.8)	22 (46.8)	19 (40.4)
Niger						
2014–2015	3503	1954 (55.8)	663 (33.9)	583 (87.9)	72 (10.9)	8 (1.2)
2015–2016	1798	1301 (72.4)	268 (20.6)	226 (84.3)	33 (12.3)	9 (3.4)
2016–2017	3088	1933 (62.6)	686 (35.5)	607 (88.5)	63 (9.2)	16 (2.3)
Togo						
2014–2015	73	68 (93.2)	32 (47.1)	20 (62.5)	12 (37.5)	0 (0)
2015–2016	291	273 (93.8)	80 (29.3)	67 (83.8)	12 (15.0)	1 (1.3)
2016–2017	441	249 (56.5)	134 (53.8)	112 (83.6)	21 (15.7)	1 (0.8)
**Total (all countries and seasons)**	**17 237**	**11 721 (68.0)**	**4018 (34.3)**	**2266 (56.4)**	**1586 (39.48)**	**165 (4.1)**

PCR or culture was performed at an NRL on specimens from 68% (11 721 of 17 237) of suspected cases overall, with proportions of cases tested ranging from 55.8% in Niger during the 2014–2015 season to 96.7% in Chad during the 2016–2017 season. Across all countries and seasons, a causative pathogen was confirmed in 34.3% of tested case specimens (4018 of 11 721), with the confirmation percentage ranging from 20.6% in Niger during the 2015–2016 season to 53.8% in Togo during the 2016–2017 season. The greatest number of seasonal reported cases in our data set occurred in Niger during the 2014–2015 meningitis season (3503 suspected and 664 confirmed cases among MenAfriNet districts), which experienced an epidemic of *N. meningitidis* serogroup C ST-10217 [17].

**Figure 2.**
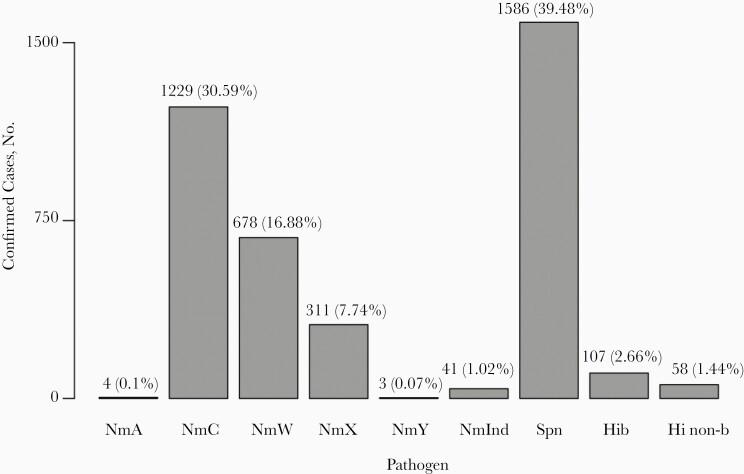
Confirmed bacterial meningitis cases by pathogen. Bar heights represent numbers of confirmed meningitis cases associated with each bacterial pathogen, pooled across all 12 analyzed country-seasons. Abbreviations: Hi non-b, *Haemophilus influenzae* non–type b; Hib, *Haemophilus influenzae* type b; NmA, NmC, NmW, NmX, and NmY, *Neisseria meningitidis* serogroups A, C, W, X, and Y, respectively; NmInd, indeterminate/unknown serogroup of *N. meningitidis*; Spn, *Streptococcus pneumoniae*.

In all country-seasons, *N. meningitidis* or *S. pneumoniae* was the most common bacterial species identified in specimens from confirmed cases [Table 1]. Cases attributable to *H. influenzae* were comparatively uncommon, comprising only 4.1% (n = 165) of all confirmed cases; 65.0% of these were *H. influenzae* type b (n = 107). The most common serogroup among the 2266 confirmed *N. meningitidis* cases was C (54.2%, n = 1229) followed by W (29.9%, n = 678) and X (13.7%, n = 311) (Figure 2). Serogroups A and Y were detected in only 4 and 3 cases, respectively, and no cases of confirmed serogroup B disease were observed. Serogroup could not be determined for 1.8% (n = 41) of confirmed meningococcal meningitis cases. Only 1 confirmed case of group B *Streptococcus* meningitis was reported, in the 2015–2016 Mali season.

### Estimating the Relative Burden of Causative Pathogens

**Figure 3.**
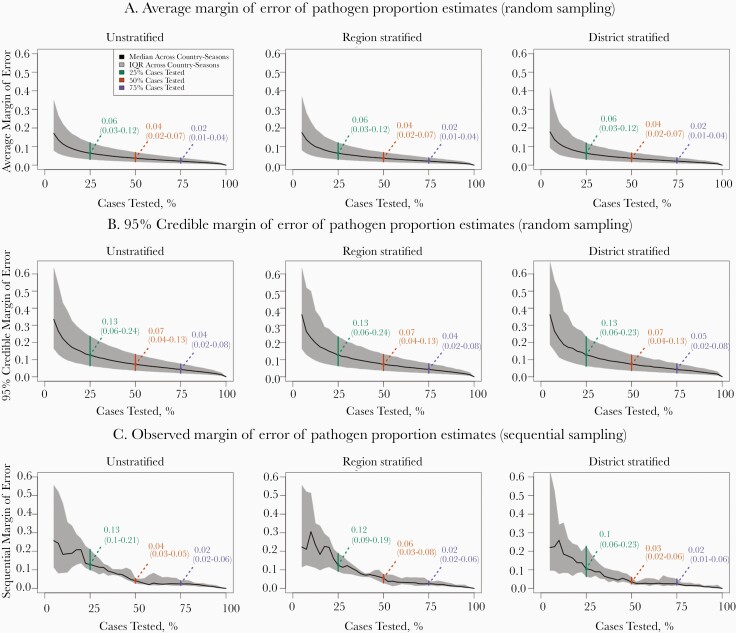
Margin of error of pathogen proportion estimates by testing coverage. When a sample of tested cases is used to estimate the true share of bacterial meningitis cases caused by each pathogen (the pathogen proportion), the margin of error for the sample is defined as the greatest pathogen-specific absolute error, such that the estimates for all pathogens are within the margin of error of the true proportion. Thus, lower margins of error indicate more accurate and precise sets of estimates. In this figure, we show how different levels of geographic stratification (*columns*) and testing coverage (*x-axis*) affect the average (*A*) and 95% credible (*B*) margin of error under random sampling, and the observed margin of error (*C*) under sequential sampling. These margin of error values (*y-axis*) are calculated individually for each of the 12 country-seasons, and summarized in terms of the median (*black lines*) and interquartile range (IQR; *shaded regions*); distinct curves for each country-season can be found in Supplementary Figure 1). Green, orange, and purple text denote specific median (IQR) margin of error values when 25%, 50%, and 75% of cases are tested, respectively.

To understand the trade-off between accurately understanding bacterial meningitis epidemiology and conserving resources by limiting laboratory testing, we compared pathogen proportion estimates generated from subsets of tested cases to the true proportions generated from the full set of tested cases in each country-season. Figure 3 shows distributions of the margin of error of pathogen proportion estimates at various levels of testing. When random, unstratified sampling is performed in the median (interquartile range [IQR]) country-season, pathogen proportion estimates have an average margin of error (the greatest absolute error across pathogens) of 0.06 (0.03–0.12), 0.04 (0.02–0.07), and 0.02 (0.01–0.04) when 25%, 50%, and 75% of cases are tested, respectively. That is, when half of cases are randomly selected for testing in the median country-season analyzed, the most erroneous pathogen proportion estimate has an absolute error of 0.04, on average. 

At low levels of testing coverage, randomly testing cases over the entire duration of the season generally produced more accurate pathogen proportion estimates than sequentially testing the first observed cases; testing the first 25%, 50%, and 75% of cases in a country-season produces median sequential margin of error values of 0.13 (IQR, 0.10–0.21), 0.04 (0.03–0.05), and 0.02 (0.02–0.06), respectively.

We also calculated a 95% credible margin of error across the 2000 subsets of cases generated through random sampling. In the median country-season analyzed, randomly testing 25%, 50%, and 75% of cases without geographic stratification was associated with 95% credible margins of error of 0.13 (IQR, 0.06–0.23), 0.07 (0.04–0.13), and 0.04 (0.02–0.08), respectively. That is, when half of cases are selected for testing in the median country-season analyzed, the most erroneous pathogen proportion estimate has an absolute error ≤0.07 in 95% of random samples. These median 95% credible margin of error values are over twice as high as the associated medians of the average margin of error. Random sampling with geographic stratification, either by region or health district, did not produce meaningfully different values of the average, sequential, or 95% credible margins of error.

Regardless of the sampling method or level of geographic stratification, we observed that the country-seasons with the least precise estimates of relative pathogen burden were those in which there were relatively few cases with an available laboratory testing result (Supplementary Figure 1 and Supplementary Table 1). In the 6 analyzed country-seasons in which between 68 and 273 cases were tested, the median margin of error values were consistently over twice as high as those from the 6 analyzed country-seasons in which between 1301 and 1954 cases had a laboratory test result. This observation was expected (refer to Supplementary Appendix 1 for more details) and demonstrates that testing of a higher proportion of case specimens is needed to accurately understand bacterial meningitis epidemiology in the context of a lower burden of disease.

### Estimating the Proportion of Tested Cases Confirmed as Bacterial Meningitis

**Figure 4.**
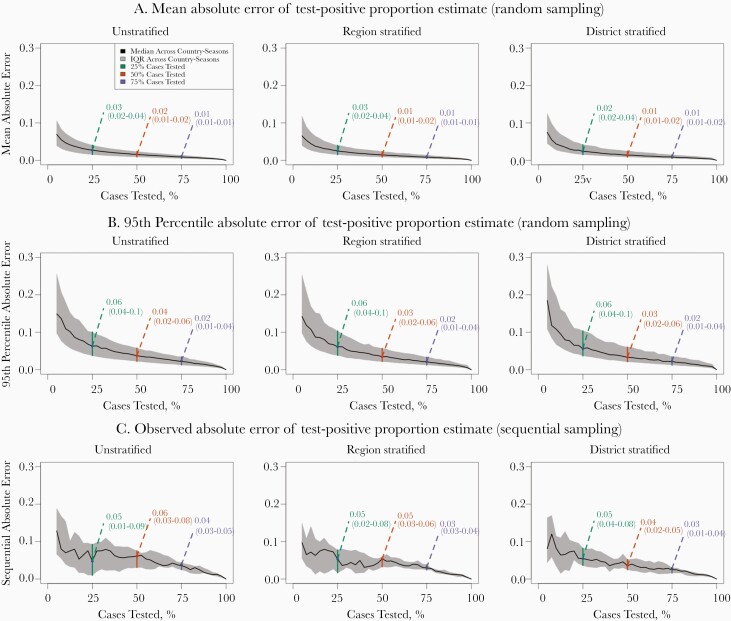
Margin of error of test-positive proportion estimates by testing coverage. We define the test-positive proportion as the proportion of tested suspect meningitis cases which were confirmed as bacterial meningitis. Figure shows how different levels of geographic stratification (columns) and testing coverage (*x-axis*) affect the mean (*A*) and 95th percentile (*B*) absolute error under random sampling, and the observed absolute error (*C*) under sequential sampling, of the test-positive proportion. These absolute error values (*y-axis*) are calculated individually for each of the 12 country-seasons, and summarized in terms of the median (*black lines*) and interquartile range (IQR; *shaded regions*). Green, orange, and purple text denote specific median (IQR) absolute error values when 25%, 50%, and 75% of cases are tested, respectively.

The proportion of tested cases that were confirmed as bacterial meningitis (the “test-positive proportion”) is an important surveillance indicator used to understand bacterial meningitis disease burden as well as surveillance system functioning for a given location and period of time [12]. This proportion has a fixed value for a given location and time period when all suspect cases are tested, but is subject to variability when random subsets of cases are selected for testing (Figure 4). When random, unstratified sampling is performed in the median country-season analyzed, the estimated test-positive proportion has a mean absolute error of 0.03 (IQR, 0.02–0.4), 0.02 (0.01–0.02), and 0.01 (0.01–0.01), and a 95th percentile absolute error of 0.06 (0.04–0.10), 0.04 (0.02–0.06), and 0.02 (0.01–0.04), when 25%, 50%, and 75% of cases are tested, respectively. That is, when half of cases are randomly selected for testing in the median country-season analyzed, the estimated test-positive proportion differs from the true value by 0.02 in the average sample, and differs by ≤0.04 in 95% of samples. 

Sequentially selecting cases over the observation period produced the least accurate test-positive proportion estimates: in the median country-season analyzed, the observed absolute error of the test-positive proportion is 0.05 (IQR, 0.01–0.09), 0.06 (0.03–0.08), and 0.04 (0.03–0.05) when the first 25%, 50%, and 75% of cases are sequentially selected for testing without geographic stratification. Stratifying the sampling process by region or district did not produce meaningfully different results.

### Case Study: Detecting Rare NmA Cases in the Burkina Faso 2014–2015 Season

**Figure 5.**
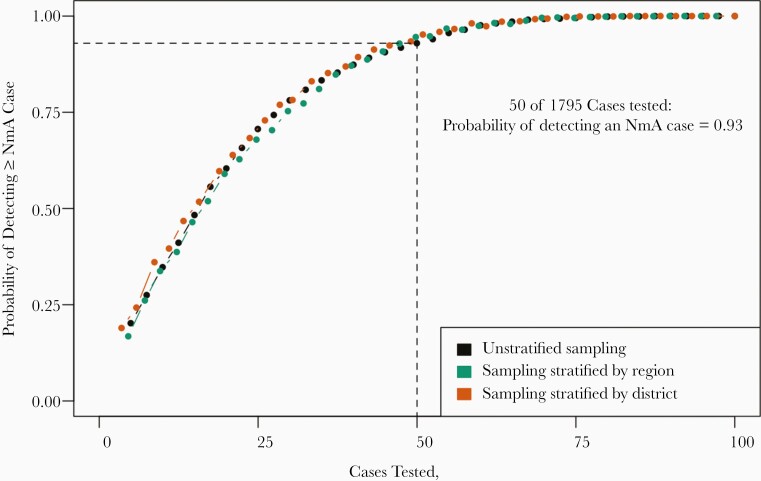
Probability of detecting a rare *Neisseria meningitidis* serogroup A (NmA) case by testing coverage, Burkina Faso, 2014–2015 season.

During the 2014–2015 meningitis season in Burkina Faso, a total of 2454 suspected cases were reported. Laboratory testing was performed on specimens from 1795 (73.1%) of these cases, resulting in the confirmation of 4 NmA cases. Given the importance of detecting rare NmA cases via surveillance, as they provide evidence of NmA transmission, we evaluated the probability of detecting ≥1 of these NmA cases under different random testing strategies. With random unstratified samples of 25%, 50%, and 75% of tested cases, the probabilities of detecting ≥1 of the NmA cases were 0.71, 0.93, and 0.99, respectively. Gains in the sensitivity of random testing beyond 50% sampling were minimal, and the level of geographic stratification in the sampling process did not meaningfully affect our findings [Figure 5].

## Discussion

This analysis explored the accuracy of pathogen proportion estimates from meningitis case-based surveillance at different levels of testing coverage and geographic stratification in countries of the African meningitis belt. Our findings suggest that in approximately three-quarters of country-seasons, testing a random sample of 50% of suspected meningitis cases would be sufficient to estimate the true percentage of cases caused by each pathogen within 7% on average, and within 13% with 95% confidence. At 50% testing coverage or higher, sampling cases sequentially from the start of the season instead of randomly did not meaningfully affect the accuracy of pathogen proportion estimates in the country-seasons analyzed. This suggests that even with nonrandom sampling, testing of specimens from ≥50% of cases would still provide reliable insight into different pathogens’ relative contributions to the burden of bacterial meningitis. The estimates of the proportion of cases caused by each pathogen were found to be more accurate during seasons with a large number of tested cases (>1300) than in seasons with relatively few (68–273) tested cases. This is consistent with our expectation that the variance of a pathogen proportion estimate is inversely related to the total number of tested cases in a country-season.

In addition to estimating the overall burden of different pathogens based on subsets drawn from each country-season, we used data from the Burkina Faso 2014–2015 season to estimate the probability of detecting ≥1 case of a rare and important pathogen at different testing levels. In this season, in which NmA was detected in 4 of 1805 tested cases, we estimated that ≥1 NmA case would have been detected 93% of the time with random sampling of 50% of suspected cases. We also found that when 50% of suspected cases are randomly selected for testing, estimates of the true prevalence of laboratory-confirmable bacterial meningitis among tested cases have a low mean absolute error of <2.5% in about 75% of country-seasons. This analysis suggests that testing approximately half of case specimens would yield both relatively accurate estimates of the distribution of causative pathogens, as well as high sensitivity to detect rare but important pathogens such as NmA.

A number of factors could cause the performance of laboratory surveillance in practice to systematically differ from the estimates we provide here. In each country-season analyzed, our sampling frame consisted of the set of tested cases in the districts covered by MenAfriNet case-based surveillance. For Burkina Faso, all districts nationwide conduct case-based surveillance; however, in the remaining countries only a subset of districts implemented meningitis case-based surveillance in the years covered in our data. Given the inverse relationship we describe between the total number of cases tested and the error of pathogen-specific burden estimates, accuracy targets could be achieved by testing a lower proportion of cases than estimated in our analysis if the sampling frame was expanded to include suspected cases in additional districts. Conversely, if the introduction of next-generation meningococcal conjugate vaccines significantly reduces the overall number of suspected bacterial meningitis cases in meningitis belt countries [18], a higher proportion of the remaining cases may need to be tested to avoid losses in accuracy.

In addition, tested cases may not be representative of all suspected cases in the country-seasons analyzed, which would affect the validity of using only tested cases as our sampling frame. Finally, because truly random testing over the course of a season is not feasible given the need to obtain timely results, estimates of relative pathogen burden may become biased, particularly when testing coverage is low, if surveillance programs do not take steps to eliminate systematic differences between tested and untested cases. Systematic sampling methods, such as testing every *n*th suspected case at each facility, can be efficient yet minimally biased alternatives to true random sampling, and are frequently used in laboratory surveillance systems for influenza [19]. Ensuring uniform surveillance across the districts of a country can also help reduce bias in the estimates of circulating pathogens. Surveillance programs may wish to create indicators to monitor the consistency of testing coverage over space and time within the season, and adjust their sampling strategy as necessary.

Strategies of preferentially testing specimens that are more likely to be confirmed—eg, CSF specimens that are cloudy or purulent, or that are collected from more severe cases—could increase the sensitivity for detecting rare pathogens and the precision of pathogen proportion estimates. However, this form of selection could also bias pathogen proportion estimates if the features used to prioritize specimens for testing are more common in cases caused by some pathogens than others. The test-positive proportion would also be inflated under preferential testing, and would no longer represent the positive predictive value of the suspected case definition for bacterial meningitis.

In addition to the above limitations, MenAfriNet case-based surveillance was initiated in 2014, which meant that data were only available for 12 country-seasons. This limited our ability to evaluate how surveillance performance is affected by additional factors such as the number of dominant circulating pathogens, spatial differences in pathogen burden between regions and districts, and temporal changes in epidemiology over the course of the season.

For this analysis, our objective was to assess the ability of case-based meningitis surveillance to accurately estimate the true distribution of causative meningitis pathogens, as retrospectively assessed at the end of a given meningitis season, when a reduced proportion of patient specimens are collected and tested,. We did not consider the testing coverage needed to adequately perform other objectives of laboratory surveillance, such as early detection of outbreaks and epidemics and rapid determination of the causative pathogen(s), identification of differences in pathogen burden between geographic areas or periods of time within a season, or monitoring more detailed data such as antibiotic resistance or the spread of specific strains. By quantifying how discrepant subset-based estimates are from “true” values based on the full set of tested cases, future analyses could adapt our modeling framework to estimate the performance of laboratory surveillance for additional objectives.

Case-based meningitis surveillance is highly valuable to develop a comprehensive understanding of bacterial meningitis epidemiology that can be used to evaluate current public health interventions and inform policy and vaccine development. However, it is also expensive and challenging in resource-limited settings. If an accurate understanding of meningitis etiology can be gained with a lower specimen collection and testing target, this could reduce the overall burden and resources required for specimen collection, transport, and testing and improve the efficiency of the surveillance system. Our estimates of the accuracy of pathogen proportion estimates generated from subsets of cases, when considered alongside the goals of the surveillance program and resource availability, can be used to set targets for the proportion of case specimens that require confirmatory testing during routine and epidemic meningitis seasons in the meningitis belt. Preliminary findings from this analysis were used to update the 2018 World Health Organization standard operating procedures for meningitis surveillance in Africa [13]. However, our findings also demonstrate that it will be important to revisit this surveillance guidance if the introduction of new vaccines or other interventions leads to substantial reductions in the burden of bacterial meningitis in this region.

## Supplementary Data

Supplementary materials are available at *The Journal of Infectious Diseases* online. Consisting of data provided by the authors to benefit the reader, the posted materials are not copyedited and are the sole responsibility of the authors, so questions or comments should be addressed to the corresponding author.

jiab154_suppl_Supplementary_Materials

## Notes

***Acknowledgments.*** The MenAfriNet Consortium is an international consortium led and implemented by Ministère de la Santé du Burkina Faso, Ministère de la Santé et de l’Hygiène Publique du Mali, Ministère de la Santé Publique du Niger, Ministère de la Santé Publique du Tchad, Ministère de la Santé et de la Protection Sociale du Togo, Agence de Médecine Préventive, the Centers for Disease Control and Prevention, and the World Health Organization, with support and collaboration from other international and nongovernmental organizations. The authors thank all MenAfriNet partners, including participating national health systems, health centers, and laboratories.

***Supplement sponsorship.*** This supplement is sponsored by the World Health Organization and the U. S. Centers for Disease Control and Prevention.

***Financial support.*** This work was supported by the MenAfriNet Consortium through a grant from the Bill & Melinda Gates Foundation (grant OPP1084298).

***Potential conflicts of interest.*** All authors: No reported conflicts. All authors have submitted the ICMJE Form for Disclosure of Potential Conflicts of Interest. Conflicts that the editors consider relevant to the content of the manuscript have been disclosed.
