# Supplementary material for: Modeling Optimal Laboratory Testing Strategies for Bacterial Meningitis Surveillance in Africa
Source: J Infect Dis. 2021 Sep 1;224(Suppl 3):S218–27. doi: 10.1093/infdis/jiab154 (PMC8409536; doi:10.1093/infdis/jiab154)
Supplement: jiab154_suppl_Supplementary_Materials [file jiab154_suppl_supplementary_materials.docx]

Supplement:

Supplementary Figures and Tables:

Supplementary Table 1: Median and IQR of expected, observed, and 95% credible Margin of Error of Pathogen-Proportion Estimates by Testing Coverage, Total Number of Cases in Country-Season, and Level of Geographic Stratification

| **Random Sampling: Median (IQR) Average Margin of Error^a^** | | | | |
| --- | --- | --- | --- | --- |
| Total Cases with a Laboratory-Testing Result | Testing Coverage | Unstratified Sampling | Region-stratified Sampling | District-stratified Sampling |
| 68 - 273 | 25% | 0.12 (0.09-0.13) | 0.12 (0.09-0.13) | 0.12 (0.09-0.13) |
|  | 50% | 0.07 (0.05-0.07) | 0.07 (0.05-0.08) | 0.07 (0.05-0.07) |
|  | 75% | 0.04 (0.03-0.04) | 0.04 (0.03-0.04) | 0.04 (0.03-0.04) |
| 1,301 – 1,954 | 25% | 0.03 (0.03-0.04) | 0.03 (0.03-0.04) | 0.03 (0.03-0.04) |
|  | 50% | 0.02 (0.02-0.02) | 0.02 (0.02-0.02) | 0.02 (0.02-0.02) |
|  | 75% | 0.01 (0.01-0.01) | 0.01 (0.01-0.01) | 0.01 (0.01-0.01) |
| **Sequential Sampling: Median (IQR) Sequential Margin of Error^b^** | | | | |
| Total Cases with a Laboratory-Testing Result | Testing Coverage | Unstratified Sampling | Region-stratified Sampling | District-stratified Sampling |
| 68 - 273 | 25% | 0.21 (0.13-0.29) | 0.19 (0.13-0.22) | 0.23 (0.17-0.25) |
|  | 50% | 0.06 (0.04-0.09) | 0.08 (0.06-0.10) | 0.07 (0.04-0.10 |
|  | 75% | 0.05 (0.03-0.07) | 0.05 (0.03-0.06) | 0.05 (0.03-0.06) |
| 1,301 – 1,954 | 25% | 0.11 (0.09-0.13) | 0.11 (0.09-0.13) | 0.08 (0.04-0.09) |
|  | 50% | 0.03 (0.02-0.04) | 0.02 (0.01-0.05) | 0.02 (0.01-0.03) |
|  | 75% | 0.02 (0.01-0.02) | 0.02 (0.01-0.02) | 0.02 (0.01-0.02) |
| **Random Sampling: Median (IQR) 95% Credible Margin of Error^c^** | | | | |
| Total Cases with a Laboratory-Testing Result | Testing Coverage | Unstratified Sampling | Region-stratified Sampling | District-stratified Sampling |
| 68 – 273 | 25% | 0.24 (0.18-0.28) | 0.24 (0.18-0.27) | 0.24 (0.18-0.29) |
|  | 50% | 0.14 (0.10-0.16) | 0.14 (0.10-0.15) | 0.13 (0.10-0.17) |
|  | 75% | 0.08 (0.06-0.09) | 0.08 (0.06-0.09) | 0.08 (0.06-0.10) |
| 1,301 – 1,954 | 25% | 0.06 (0.06-0.07) | 0.06 (0.06-0.07) | 0.06 (0.06-0.07) |
|  | 50% | 0.04 (0.04-0.04) | 0.04 (0.04-0.04) | 0.04 (0.03-0.04) |
|  | 75% | 0.02 (0.02-0.02) | 0.02 (0.02-0.02) | 0.02 (0.02-0.02) |

^a^ Margin of error: the greatest absolute error from the true proportion among all pathogen-proportion estimates in the subset of cases. Average margin of error: the average of the margin of error estimates across 2,000 randomly selected subsets of cases.

^b^ Sequential margin of error: The observed margin of error in a subset of cases selected sequentially from the start of the season.

^c^ 95% Credible margin of error: The 95^th^ percentile margin of error value across 2,000 randomly selected subsets of cases.

Supplementary Figure 1: Margin of Error of Pathogen-Proportion Estimates by Testing Coverage, Grouped by Total Number of Cases in Season

*Each line represents one of the 12 country-seasons analyzed, with the color of the line indicating the total number of cases in the country-season with an available laboratory testing result in that country-season.*


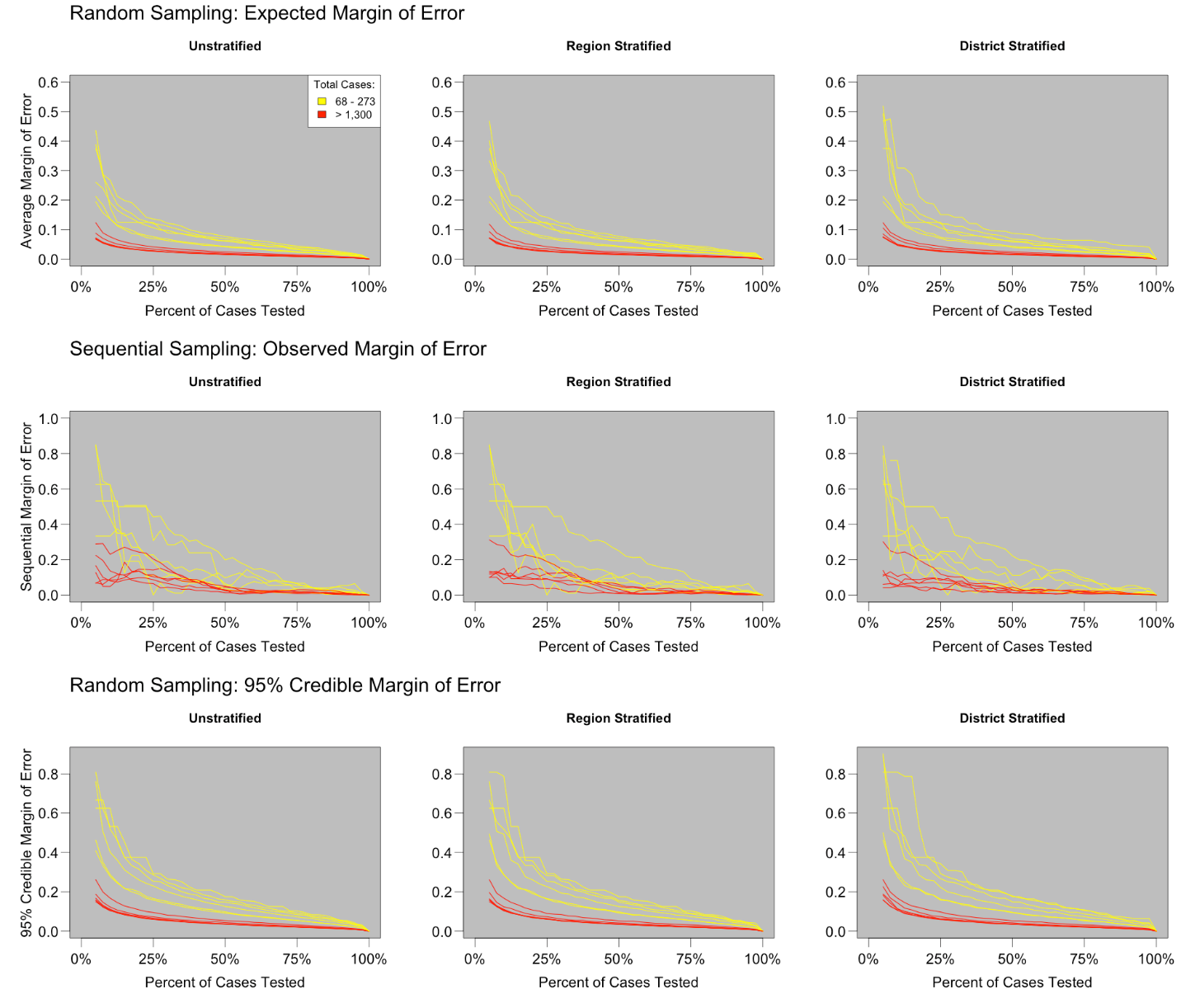


Supplementary Figure 2: Variance of Pathogen-Proportion Estimates by Number of Cases, Derived from the Multivariate Hypergeometric Distribution

*This figure shows the relationship between the total number of suspected meningitis cases in a country-season and the variance of any given pathogen’s attributable case proportion estimate as a function of the true proportion, P. This relationship is shown for different values of the percent of confirmable cases selected for testing: 25%, 30%, and 35%, and assumes that 50% of tested suspect cases will be confirmed. Variance values were calculated using a modified version of the multivariate hypergeometric distribution, and assume random sampling (Supplementary Appendix 1). This figure demonstrates that when pathogen-proportion values and sampling levels are held constant, more precise estimates of relative pathogen burden can be expected when the overall volume of cases in a country-season is higher, with estimates from country-seasons with fewer than 1,000 confirmable cases being particularly variable.*

*
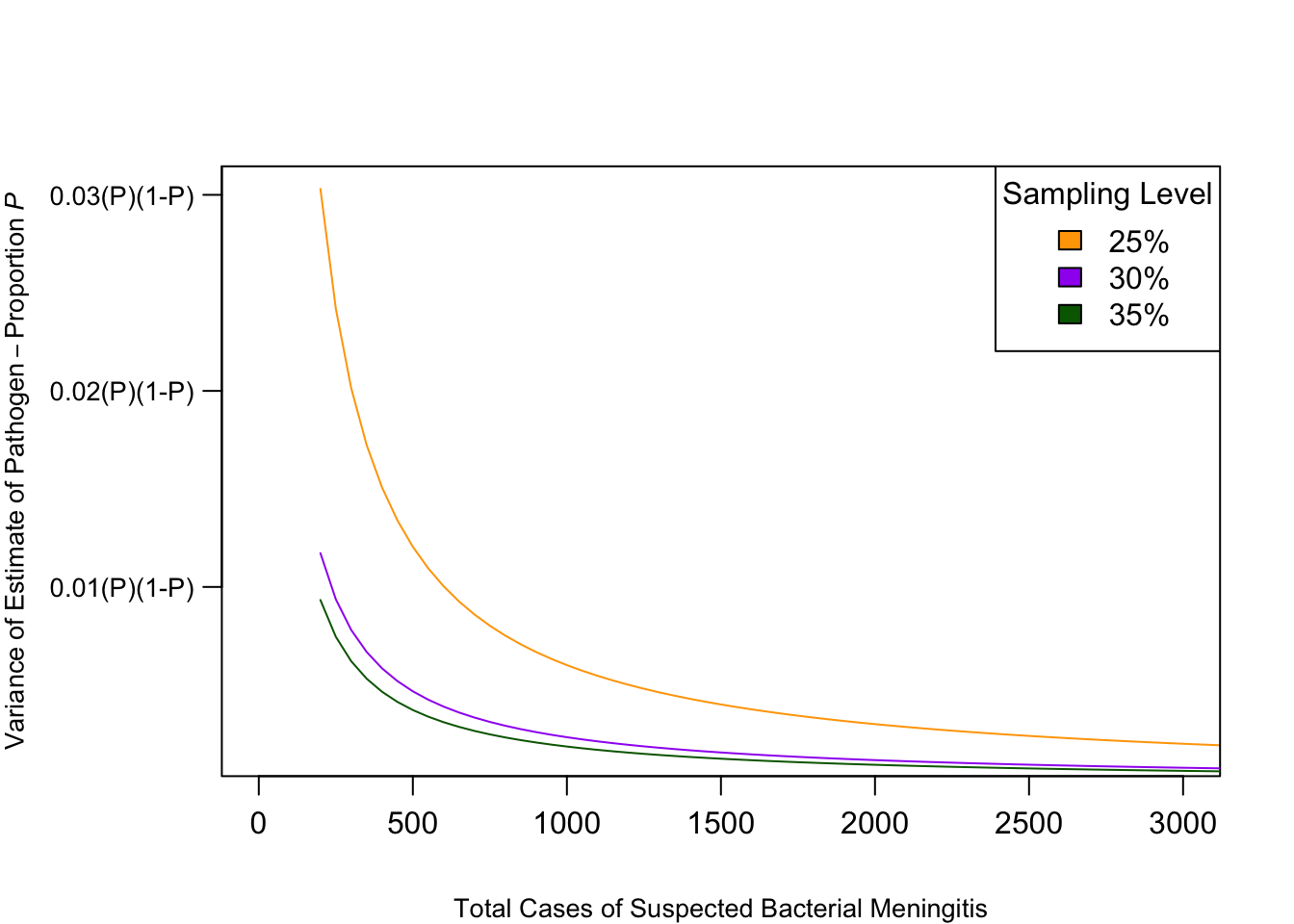
*

Appendix 1:

We observed that for a given sampling strategy, pathogen-proportion estimates in country-seasons with relatively few cases tend to be less accurate (have higher expected margins of errors) than those from country-seasons with a larger pool of cases (Supplementary Figure 1, Supplementary Table 1). Here, we show how this pattern is consistent with statistical expectations.

In the context of our analysis, when there is a population of $N= \sum_{i=1}^{k} X_{i}$ total cases caused by $k$ pathogens, with each pathogen $i$ responsible for $X_{i}$ cases, we can use the multivariate hypergeometric distribution to model $P(x_{i})$, the probability of selecting $x_{i}$ cases of pathogen $i$ when drawing a random sample of size $n= \sum_{i}^{k} x_{i}$.

By setting the population and sample proportion of cases caused by pathogen $i$ equal to $P_{i}= X_{i} / N$ and $p_{i}= x_{i} / n$ respectively, and defining the testing level as the proportion $t=\frac{n}{N}$, we can express the multivariate hypergeometric distribution in terms of pathogen-proportions. Under this modified distribution, in which most parameters are expressed in terms of proportions rather than raw counts, the expected value of pathogen-proportion estimates $p_{i}$ is equal to their population value $P_{i}$. The function for variance in the modified distribution. $V\left( p_{i} \right)= \frac{P_{i}(1-P_{i})(1-t)}{t(N-1)}$, is derived by substituting $P_{i}$ for $X_{i} / N$ and $tN$ for $n$ into the variance function of the original multivariate hypergeometric distribution, $V\left( x_{i} \right)= \frac{X_{i}(1-\frac{X_{i}}{N})n(N-n)}{N(N-1)}$ , multiplying by $n^{-2}$ ($x_{i}$ is converted to $p_{i}$ by multiplying by $n^{-1}$. When a random variable ($x_{i}$) is multiplied by a constant ($n^{-1}$), the variance of the new product is found by multiplying the variance of the original random variable by the square of the constant ($n^{-2}$)), and simplifying.

Notably, the variance $V\left( p_{i} \right)$ is inversely proportional to the size of the sampling frame $N$, the total population of cases from which a sample are selected for testing. Thus, if the testing level and true pathogen-proportion values are held constant, we would expect estimates of relative pathogen burden to be less precise when sampling from a country-season with fewer cases (Supplementary Figure 2).
